# Supplementary material for: Characterization and Evaluation of Bone-Derived Nanoparticles as a Novel pH-Responsive Carrier for Delivery of Doxorubicin into Breast Cancer Cells
Source: Int J Mol Sci. 2020 Sep 14;21(18):6721. doi: 10.3390/ijms21186721 (PMC7555837; doi:10.3390/ijms21186721)
Supplement: Supplementary file 1 [file ijms-21-06721-s001.pdf]

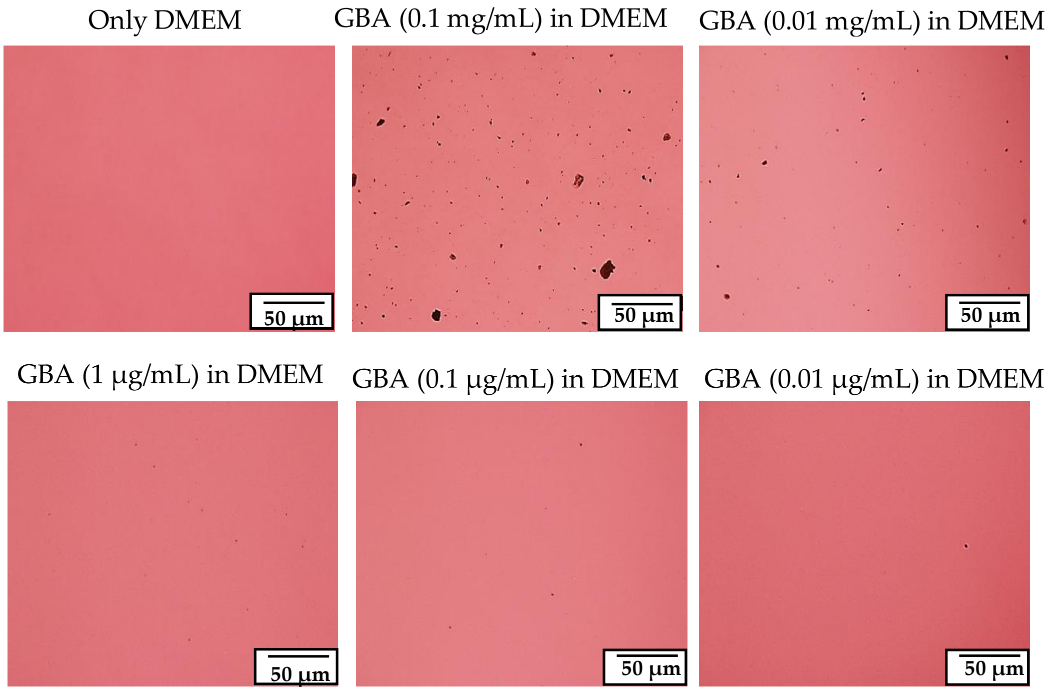

Figure S1. Optical microscopic images of different concentrations (0.1 mg/mL, 0.01 mg/mL, 1.00 µg/mL, 0.10 µg/mL and 0.01 µg/mL) of GBA in DMEM (pH 7.4). All images were taken after 30 min incubation at 37 °C. Magnification of 10 × and a scale bar of 50 µm were used.

1 µg/mL GBA Suspension

			Size (d.nm):	% Intensity:	St Dev (d.nm):
Z-Average (d.nm):	217.6	Peak 1:	410.1	53.0	94.73
PdI:	0.415	Peak 2:	6.629	16.8	1.298
Intercept:	0.971	Peak 3:	21.44	16.1	4.706
Result quality :	Refer to quality report				

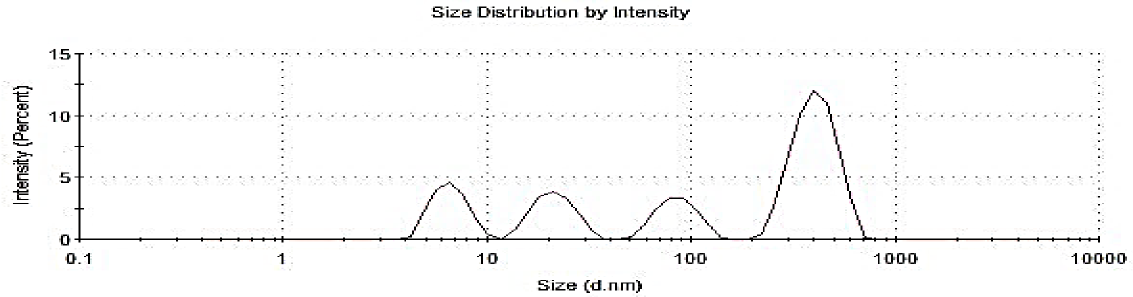

0.01 mg/mL GBA Suspension

			Size (d.nm):	% Intensity:	St Dev (d.nm):
Z-Average (d.nm):	478.2	Peak 1:	405.3	86.6	115.7
PdI:	0.430	Peak 2:	4978	13.4	618.9
Intercept:	0.248	Peak 3:	0.000	0.0	0.000
Result quality :	Refer to quality report				

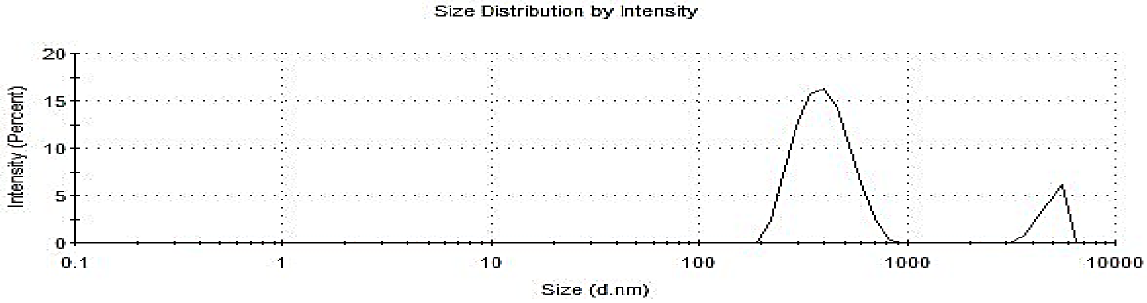

0.1 mg/mL GBA Suspension

			Size (d.nm):	% Intensity:	St Dev (d.nm):
Z-Average (d.nm):	525.9	Peak 1:	290.1	96.6	67.79
PdI:	0.530	Peak 2:	5560	3.4	0.000
Intercept:	0.248	Peak 3:	0.000	0.0	0.000
Result quality :	Refer to quality report				

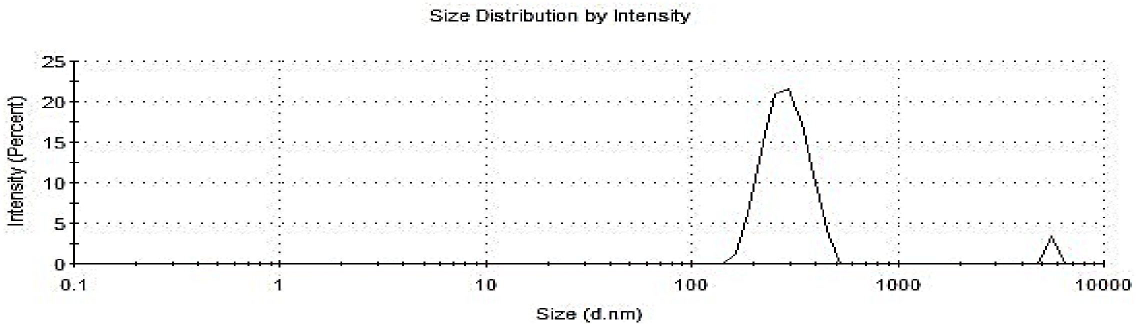

1 mg/mL GBA Suspension

			Size (d.nm):	% Intensity:	St Dev (d.nm):
Z-Average (d.nm):	938.7	Peak 1:	735.1	85.5	160.5
PdI:	0.652	Peak 2:	151.6	14.5	21.57
Intercept:	0.912	Peak 3:	0.000	0.0	0.000
Result quality :	Refer to quality report				

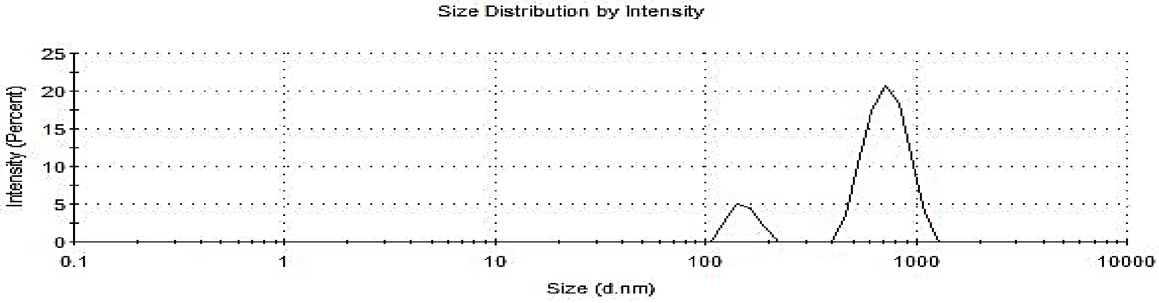

10 mg/mL GBA Suspension

			Size (d.nm):	% Intensity:	St Dev (d.nm):
Z-Average (d.nm):	1336	Peak 1:	1032	89.8	311.2
PdI:	0.390	Peak 2:	5229	10.2	458.0
Intercept:	0.854	Peak 3:	0.000	0.0	0.000
Result quality :	Good				

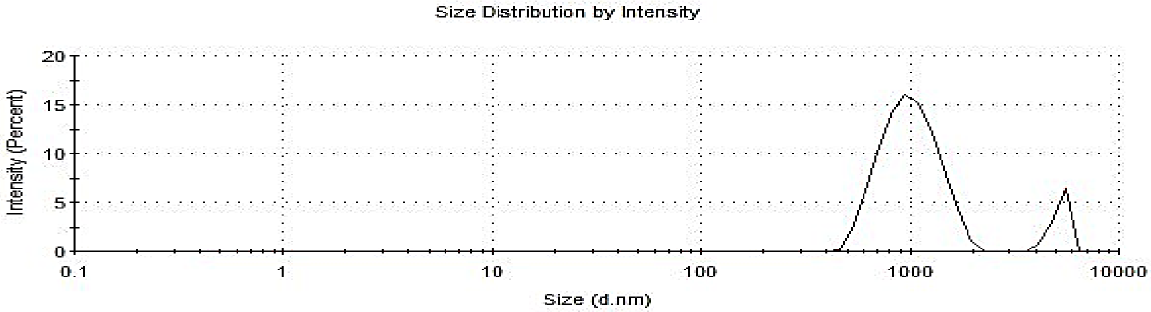

100 mg/mL GBA Suspension

			Size (d.nm):	% Intensity:	St Dev (d.nm):
Z-Average (d.nm):	2371	Peak 1:	1182	94.8	217.1
PdI:	0.527	Peak 2:	274.9	5.2	26.85
Intercept:	0.939	Peak 3:	0.000	0.0	0.000
Result quality :	Refer to quality report				

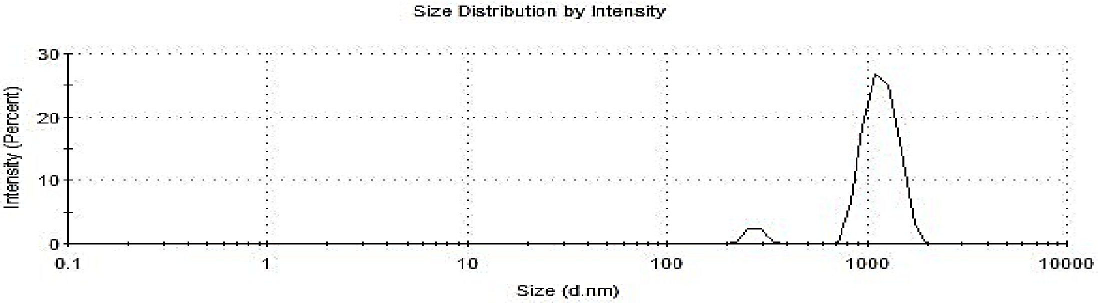

Figure S2. Representative data of particle size distribution by intensity for different concentrations (1 µg/mL, 0.01 mg/mL, 0.10 mg/mL, 1.00 mg/mL, 10.00 mg/mL and 100.00 mg/mL) of GBA suspension in water.

A. FESEM Micrographs of GBA Powder at Low Resolution

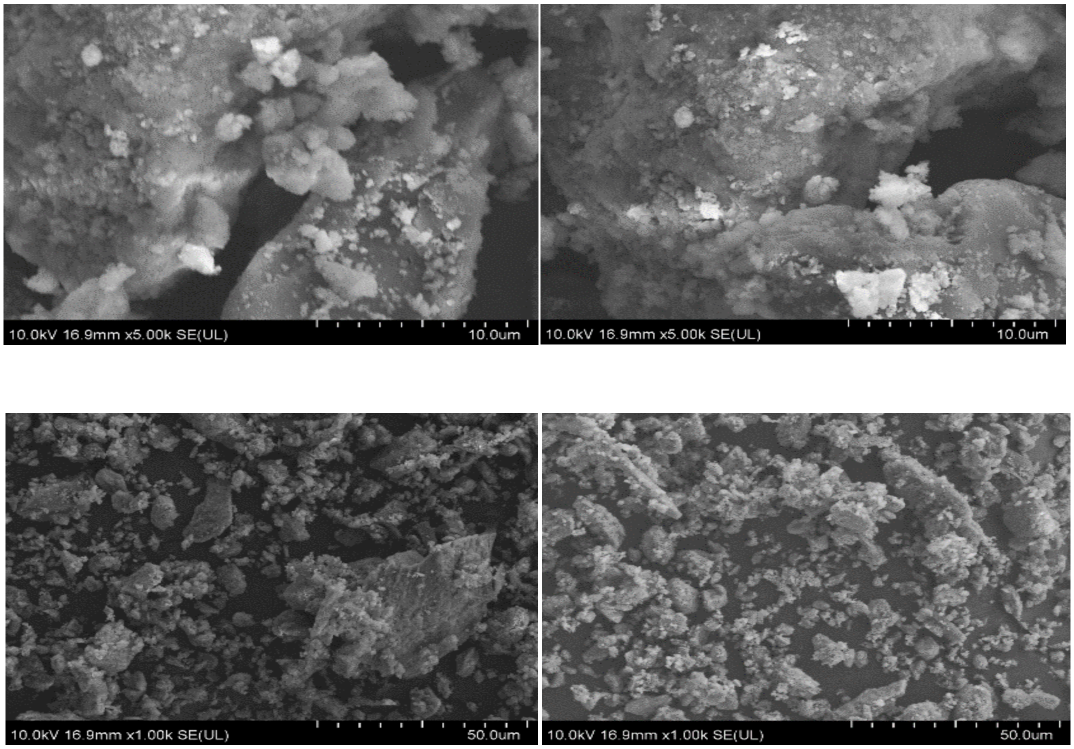

B. FESEM Micrographs of GBA Powder at High Resolution

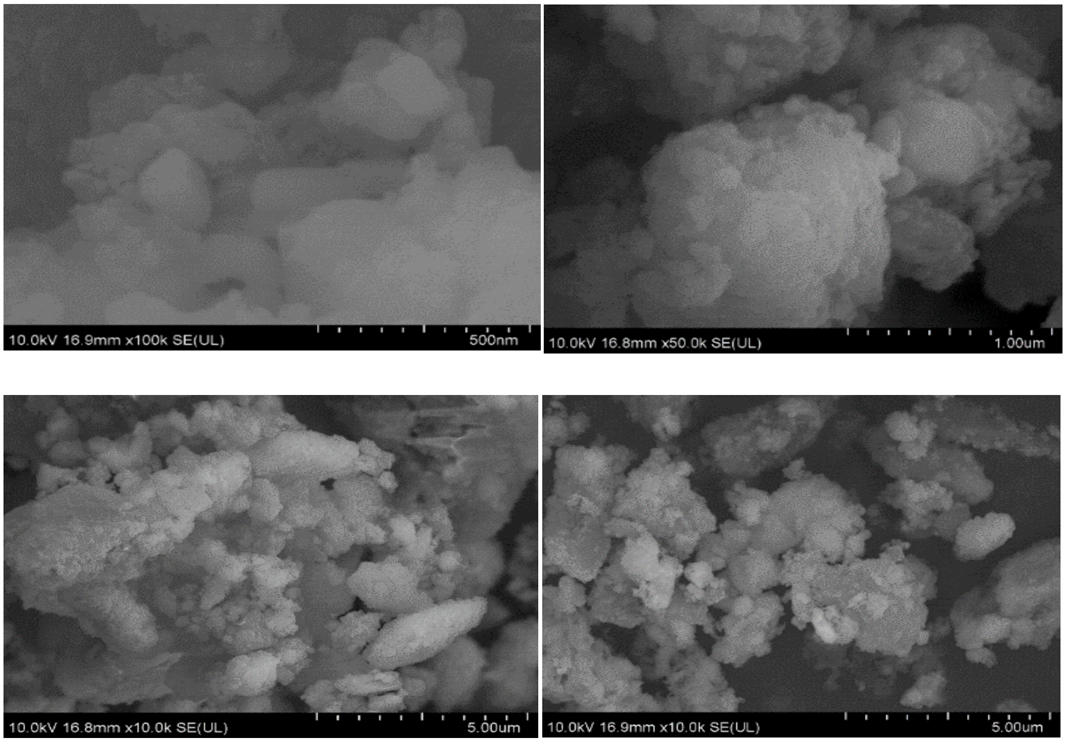

Figure S3. Low and high resolution FESEM images of pristine GBA powder. (A) Low resolution FESEM micrographs of pristine GBA powder. (B) High resolution FESEM micrographs of pristine GBA powder.

A. Pellet size in water

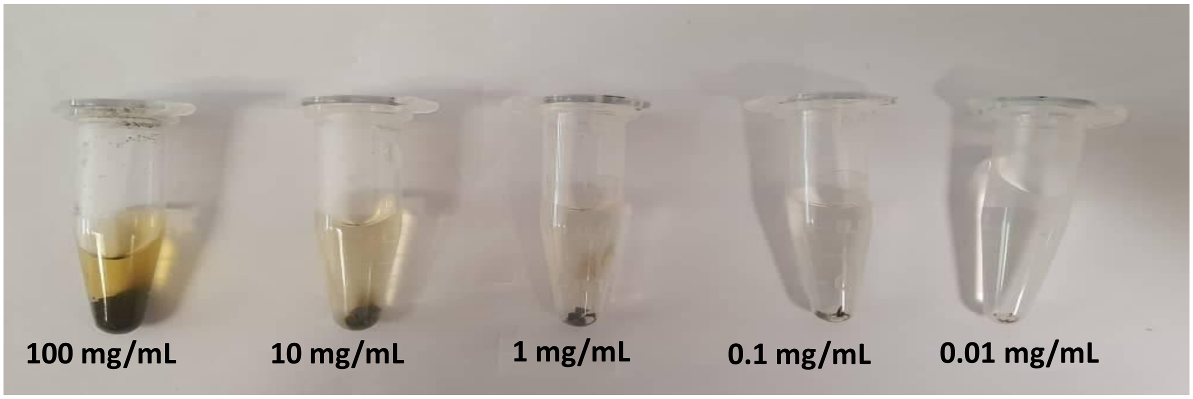

B. Pellet size in HCl

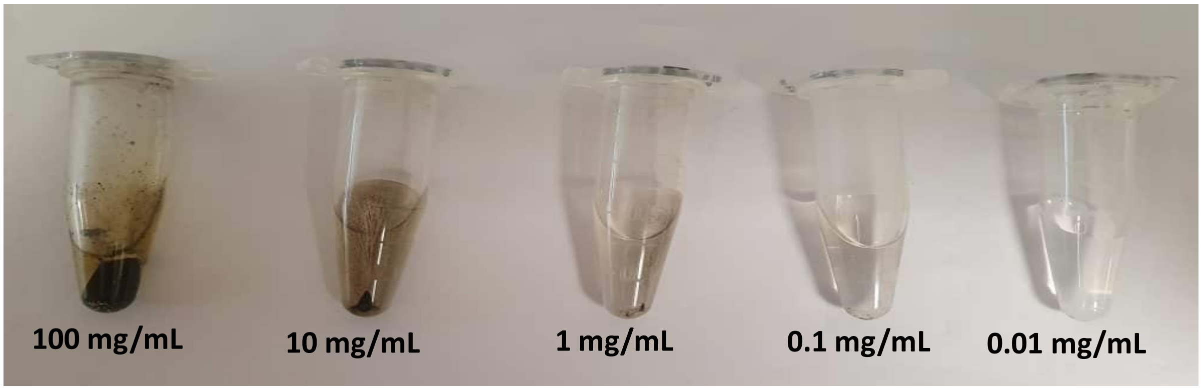

Figure S4. Size of pellets for different concentrations (100, 10, 1, 0.1 and 0.01 mg/mL) of GBA suspension in (A) water and (B) HCl (1N) after centrifugation at 13,000 rpm for 20 min.

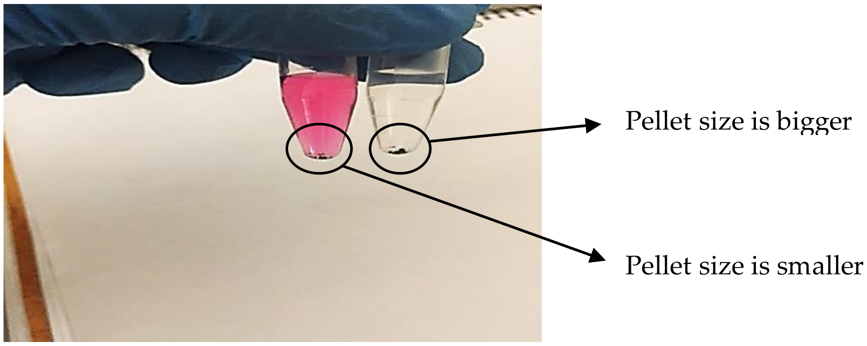

Figure S5. Solubility of GBA powder in DMEM and bicarbonated water.
